# Changes of Uterine Muscular Tissue after Parturition

**Published:** 1883-04

**Authors:** 


### Selections.

### Changes of Uterine Muscular Tissue after Parturition.

Prof. Wojtschechoffsky has examined, microscopically, ten uteri, removed from women who died within thirty-five days post partum. He offers the following as the result of his investigations:

1. The fatty degeneration of the uterine muscular tissue, upon which the diminution in size of the uterus depends, begins immediately after parturition, and covers a period of more than five weeks.

2. The degeneration attacks alike all parts of the organ (fundus, corpus and collum).

3. It could not be ascertained whether the process began at an earlier period in the outer or inner muscular layer of the uterus.

4. The interstitial tissue of the uterus does not take an active part in the process of involution.

5. The process of obliteration of the blood vessels covers a greater period than the entire involution.

6. The appearance of free pigment in the uterus is not stationary.—*Deutsche Med. Zeit.*

